# Long-Term Stability of Alveolar Bone Dimensions Using the Socket Shield Technique: A Retrospective Cone Beam Computed Tomography Analysis

**DOI:** 10.1155/ijod/3880963

**Published:** 2025-11-26

**Authors:** Massimo Natale, Roberto Lo Giudice, Francesco Puleio

**Affiliations:** ^1^Private Practitioner, Messina 98100, Italy; ^2^Department of Biomedical and Dental Sciences and Morphofunctional Imaging, Messina University 98121, Messina, Italy

**Keywords:** alveolar bone preservation, bone remodeling, buccal bone resorption, cone-beam computed tomography, implantology, partial extraction therapy, ridge preservation, socket shield technique

## Abstract

**Background:**

Alveolar bone resorption following tooth extraction, especially in the buccal plate, can compromise ridge dimensions, esthetic outcomes, and implant stability. The socket shield technique (SST) aims to preserve buccal bone by retaining a thin buccal root fragment, maintaining the periodontal ligament (PDL) and associated bone.

**Objective:**

To evaluate long-term dimensional stability of the alveolar ridge following immediate implant placement using SST, focusing on horizontal buccal and palatal bone changes over a 3-year period.

**Methods:**

This retrospective study included 20 patients requiring single-tooth implant-supported restorations in the esthetic zone, treated between 2019 and 2021 at the University of Messina. All implants were placed using SST with the “Preserving Nature” protocol, retaining a buccal root fragment of approximately 1 mm. Immediate provisionalization was performed following standardized surgical and prosthetic protocols. Cone-beam computed tomography (CBCT) scans at baseline (T0) and 3 years (T1) were aligned using a three-point superimposition method to standardize measurements. Paired statistical analysis assessed changes in bone dimensions, with significance set at *p*  < 0.05.

**Results:**

No measurable horizontal buccal bone loss was detected in any patient. Minimal horizontal palatal bone loss occurred in 15% of cases, while vertical palatal bone height remained stable. Mean dimensional changes were not statistically significant (*p*=0.109).

**Conclusion:**

SST provided excellent preservation of alveolar bone dimensions, particularly in the buccal plate, over 3 years. The technique offers predictable benefits for implant placement in the esthetic zone. Despite limitations related to retrospective design and small sample size, the standardized surgical protocol and precise CBCT analysis support reproducibility. Randomized controlled trials are warranted to confirm these results.

## 1. Introduction

Dental implant therapy is a well-established procedure for replacing missing teeth, with high success rates and predictable long-term outcomes. However, tooth extraction results in substantial alterations to the alveolar ridge, primarily due to buccal cortical bone resorption, creating challenges for subsequent implant placement and esthetic outcomes [1, 2]. Araújo et al. [3] demonstrated in an animal histological study that this resorption is linked to the loss of intraligamentary vessel supply following periodontal ligament (PDL) removal. Ridge resorption progresses rapidly in the early healing phase, with most volume loss occurring within the first year and continuing more slowly thereafter [4]. These dimensional changes reduce available bone, compromise esthetics, and often require augmentation procedures [5, 6].

The bundle bone, a histologically distinct part of the alveolar bone anchored by Sharpey's fibers, is highly dependent on tooth presence. Its resorption begins soon after extraction, particularly on the buccal aspect, driving vertical and horizontal ridge collapse—especially in the anterior maxilla where the buccal plate is thin and mainly composed of bundle bone [3]. Tan et al. [2] confirmed that about two-thirds of bone loss occurs within 3 months, initiated by bundle bone resorption. Histomorphometric and radiographic studies have reinforced the influence of bundle bone on residual ridge morphology [7, 8].

Several strategies have been developed to counteract postextraction bone loss, including socket preservation, guided bone regeneration (GBR), immediate implant placement, and, more recently, the socket shield technique (SST) [9, 10]. SST, introduced by Hürzeler et al. [9], retains a thin buccal root fragment (“shield”) while removing the palatal or lingual portion. Preserving the buccal root segment maintains the PDL and its vascular supply, potentially reducing bundle bone resorption and limiting physiological remodeling [11, 12]. Variations in SST differ in shield thickness, instrumentation, and palatal root management.

The present study adopts the standardized “Preserving Nature” protocol from the Italian Socket Shield Society (SISS). This method removes the entire palatal root, including the apical portion, and preserves only a buccal shield of about 1 mm. The aim is to achieve primary implant stability in palatal bone, minimizing shield interference and reducing risks of displacement, infection, or unwanted remodeling.

Histological and clinical evidence indicates that preserving a buccal root fragment can prevent buccal bone resorption, maintain PDL function, and support peri-implant tissues in implant-supported restorations, resulting in superior esthetic and functional outcomes compared with conventional placement [13, 14]. In post-extractive sites, SST can limit volume reduction and maintain favorable soft tissue contours.

Retrospective and prospective studies report minimal dimensional changes and excellent esthetics with SST over long-term follow-up [15–17]. A systematic review by Gharpure and Bhatavadekar [18] highlighted its promise, noting stable hard and soft tissue contours in the short to mid-term. However, additional long-term data are required to confirm stability around SST-treated implants.

This study retrospectively evaluates buccopalatal cortical bone dimensional changes around implants placed using SST, assessed with cone-beam computed tomography (CBCT) over a 3-year follow-up.

## 2. Materials and Methods

The study design is a retrospective analysis with a 3-year follow-up period.

The study adhered to ethical guidelines in line with the Helsinki declaration and was approved by the ethical committee with Approval No. 127-21 (30/09/2021, A.O.U. “G. Martino”)

### 2.1. Inclusion Criteria

• Patients requiring implant-prosthetic rehabilitation to replace hopeless teeth, who met the inclusion criteria for the use of the SST and for whom a second CBCT scan was available, performed later for unrelated diagnostic purposes.
• Only patients for whom a second CBCT scan was available—acquired for unrelated clinical indications—were retrospectively included. This approach avoided unnecessary additional radiation exposure.
• Patients treated between 2019 and 2021.

### 2.2. Exclusion Criteria

Exclusion criteria included the presence of systemic diseases affecting bone metabolism and ongoing treatments with antiresorptive medications or corticosteroids. A total of 20 patients were included in this study, all requiring the replacement of a single hopeless tooth through implant-supported prosthetic rehabilitation.

Surgical planning was based on CBCT examinations Planmeca ProMax 3D Mid unit (Planmeca Oy, Helsinki, Finland), operating with a voxel size of 200 µm, 90 kVp, 10 mA, and an exposure time of approximately 12 s. The field of view (FOV) was selected based on clinical need, typically Ø100 mm × 60 mm, ensuring high-resolution imaging of the region of interest. The Romexis imaging software (Planmeca Romexis version 6.4.1, Planmeca Oy, Helsinki, Finland) was used for scan alignment, landmark-based superimposition, and linear measurements.

All patients were treated by the same operator, following a standardized surgical protocol:

1. Separation of the root fragment using diamond burs (kit SISS Komet, Komet, Lemgo, Germany).
2. Luxation of the palatal portion of the root fragment, including the apical third.
3. Extraction of the palatal root portion.
4. Implant placement (Nobel Parallel, Nobel Biocare Services AG, Zurich, Switzerland).
5. Digital impression using Trios 3 (3Shape Trios A/S, Copenhagen, Denmark).
6. Final restoration delivered after 4 months.

**Figure 1:**
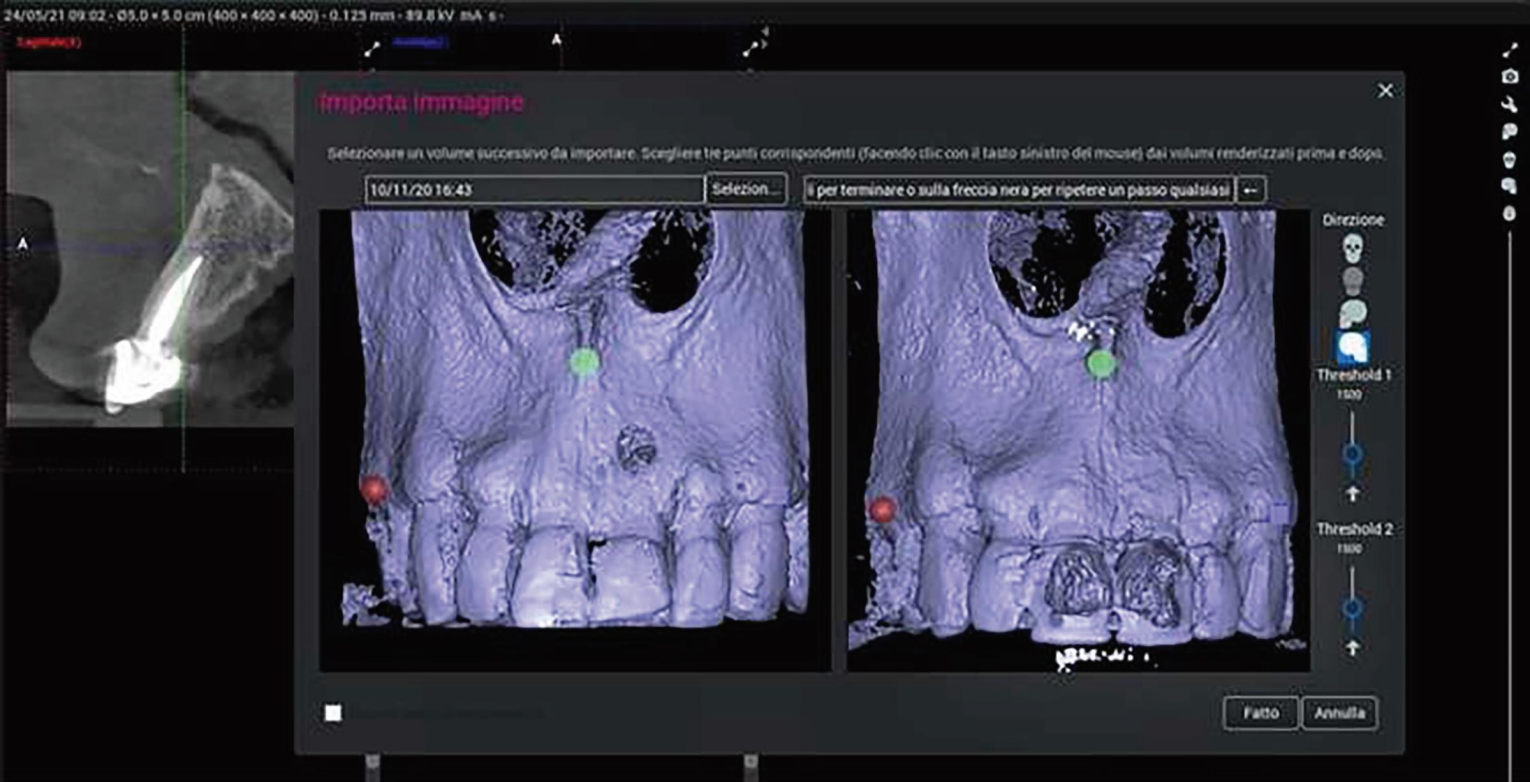
Alignment of preoperative (T0) and follow-up (T1) CBCT scans using stable anatomical landmarks (palatal vault, anterior nasal spine, and adjacent teeth) for superimposition.

**Figure 2:**
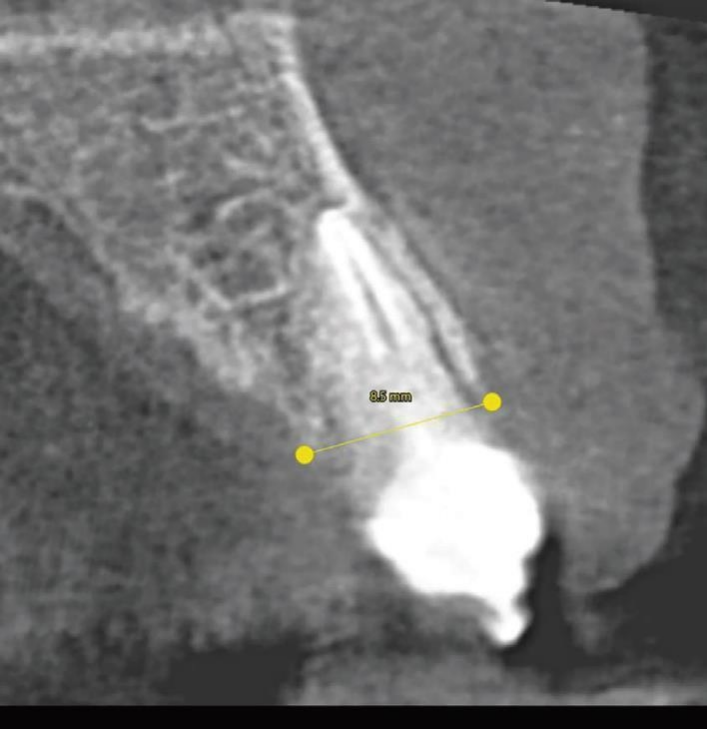
Preoperative (T0) cross-sectional CBCT view showing the measurement line drawn between buccal and palatal cortical plates at the level of the alveolar crest.

Bone thickness was measured in the bucco-palatal direction on the CBCT scan at baseline (T0) and at the 3-year follow-up (T1). Measurement standardization was ensured by applying the following protocol:

1. Registration of anatomical landmarks to allow the software to align pre and postoperative scans.
2. Identification, on the preoperative scan, of the axial section corresponding to the largest root diameter, approximating the tooth center.
3. Superimposition of pre and postoperative CBCT scans (Figure 1).
4. Drawing of a linear segment in the bucco-palatal/lingual direction to define the site for bone thickness measurement (Figure 2).
5. Remeasurement of the bone thickness at the same section identified on the preoperative scan.

**Figure 3:**
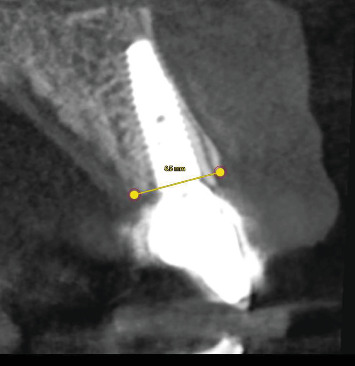
Corresponding follow-up (T1) section after three years, with repeated measurement along the identical reference line to assess dimensional changes.

Because this study was retrospective, no immediate postoperative CBCT scans were available, as all imaging data were retrieved from existing clinical records (Figure 3).

Alveolar bone dimensions were assessed using CBCT scans acquired at two time points: immediately after tooth extraction (T0) and after the healing period or at follow-up (T1). The following parameters were measured:

Alveolar thickness: the horizontal distance between the buccal (vestibular) and palatal (or lingual) cortical plates, measured at a standardized vertical level from the alveolar crest. Values were recorded in millimeters at both T0 and T1.

Horizontal buccal bone loss: defined as the difference in horizontal thickness of the buccal bone plate between T0 and T1, measured at the same level as the alveolar thickness. A reduction in thickness was considered indicative of horizontal bone resorption on the buccal aspect.

Horizontal palatal bone loss: the reduction in horizontal thickness of the palatal (or lingual) bone plate between T0 and T1, measured at the corresponding level. This parameter reflected horizontal resorption on the palatal aspect.

To enhance reproducibility and accuracy, specific anatomical landmarks used for CBCT superimposition were selected based on stable anatomical references, such as the palatal vault, anterior nasal spine, and adjacent teeth unaffected by surgical procedures. This method ensured consistent and reliable measurements across all scans. The calibration protocol for intraobserver reliability involved randomly selecting 20% of cases (four scans), which were independently reassessed by the same examiner after a 2-week interval to minimize recall bias. The intraobserver agreement (intraclass correlation coefficient [ICC] = 0.94) confirms the robustness and precision of the measurement technique.

All measurements were performed using the same imaging software, with standardized cross-sectional views to ensure reproducibility. Measurements were recorded to the nearest 0.01 mm by a calibrated examiner.

Measurement reproducibility was ensured through a dedicated software function that preserves the defined axial section and segment from the preoperative scan, allowing the follow-up scan to be assessed on the identical section. This approach enables highly accurate and reproducible comparisons of pre and postoperative bone dimensions at the exact same anatomical site.

All statistical procedures were carried out with IBM SPSS Statistics, version 29.0 (IBM Corp., Armonk, NY, USA). Normality of continuous variables (alveolar thickness at T0 and T1, vertical palatal bone height) was evaluated with the Shapiro–Wilk test, selected because it maintains good power with small samples (*n* = 20). As at least one variable departed from a normal distribution and the design involved paired observations, the nonparametric Wilcoxon signed-rank test was used to compare baseline (T0) and follow-up (T1) measurements. Continuous data are presented both as mean ± standard deviation (SD) and as median with interquartile range (IQR) to provide distribution-robust and parametric summaries.

For dichotomous outcomes—presence/absence of horizontal buccal, horizontal palatal, and vertical palatal bone loss—frequencies and percentages were calculated. Inferential testing with McNemar's *χ*^2^ was deemed inappropriate for horizontal buccal loss because all observations were zero, precluding contingency analysis. Statistical significance was set at *α* = 0.05.

All measurements were obtained by a single calibrated examiner using Romexis software. Intra-observer reliability was assessed in SPSS on a random 20% subset of scans remeasured after a 2-week interval, using a two-way random-effects ICC ([1, 2]); agreement was excellent (ICC = 0.94).

## 3. Results

The results of the measurements are presented in Tables 1, 2, and 3.

The final sample included 20 patients (12 females and 8 males; mean age 47.6 ± 8.3 years, range 34–61).

### 3.1. Interoperator Reliability

A random 20% subset (*n* = 4) was remeasured after 2 weeks; the two-way random ICC for alveolar thickness was 0.94, indicating excellent intraobserver agreement.

Overall, the mean alveolar thickness slightly decreased from 8.73 ± 1.20 mm at baseline (T0) to 8.52 ± 1.07 mm at follow-up (T1), with a mean difference of –0.21 ± 0.52 mm, which was not statistically significant (*p*=0.109). No horizontal buccal bone loss was observed in any patient, while minor palatal resorption occurred in 3 of 20 cases (15%), with a mean reduction of 0.18 ± 0.43 mm. Vertical palatal height remained stable across the entire sample.

Normality testing (Shapiro–Wilk *W* = 0.458, *p*  < 0.001) indicated a non-normal distribution; therefore, the Wilcoxon signed-rank test was applied, confirming the absence of significant dimensional changes over the 3-year period. Measurement reproducibility was excellent (ICC = 0.94), supporting the reliability of the superimposition and assessment protocol.

## 4. Discussion

The primary objective of contemporary implant-prosthetic rehabilitation is to achieve long-term functional and esthetic success. Tooth extraction initiates biological processes that alter alveolar bone structure and surrounding soft tissues, posing a major challenge in the anterior regions. Rapid postextraction remodeling, characterized by loss of bundle bone and the PDL, inevitably leads to buccal bone plate resorption and soft tissue collapse. These dimensional reductions, especially in the buccal-lingual direction, are well documented [5, 19–22].

This retrospective analysis evaluated 3-year dimensional changes in alveolar bone using CBCT following the SST. Currently, no universally accepted protocol defines key parameters, such as optimal shield thickness or instrumentation. In this study, the “Preserving Nature” protocol of the SISS was employed, involving endodontic content removal, buccal shield thinning to approximately 1 mm, and removal of the palatal root portion, including the apex. The buccal fragment was shaped to preserve the PDL to the crestal bone peaks, supporting interdental papillae. Primary stability was achieved through palatal bone anchorage, leaving the buccal shield undisturbed. Immediate provisionalization with a screw-retained PMMA crown aimed to maintain soft tissue volume and shape the emergence profile.

Inclusion was limited to patients requiring a second CBCT for unrelated clinical reasons, allowing precise pre and postoperative superimposition using Planmeca Romexis software with three anatomical reference points. This method ensured reproducible measurements on identical axial planes.

The results showed no measurable buccal bone resorption from baseline (T0) to 3-year follow-up (T1). Minimal horizontal palatal bone loss occurred in 15% of cases, with vertical palatal bone height stable. These outcomes align with pilot studies and systematic reviews demonstrating SST's ability to preserve buccal bone thickness and soft tissue contours [10, 21, 23]. Bäumer et al. [10] documented minimal remodeling with SST, while Zhang et al. [21] reported reduced bone resorption and improved esthetics compared with conventional techniques [14].

The technique applied here differs from other SST variations by systematically removing the palatal root portion, enabling palatal implant anchorage and reducing risks of shield displacement, infection, or buccal interference. The “Preserving Nature” protocol, proposed by the SISS, introduces several procedural refinements compared with international SST variants. The technique follows a “crown-down” approach, performing all shield preparation before removing the palatal root fragment to avoid contamination of the alveolar socket by rotary debris. The root is sectioned for approximately two-thirds of its canal lumen, and the palatal portion—including the apex—is removed entirely, leaving an intact buccal fragment. The shield is contoured in a characteristic “C” shape to preserve the PDL up to the crestal bone peaks, thereby maintaining interdental papillae. Its thickness is standardized to approximately 1 mm, thinner than in other published methods, enhancing predictability and minimizing internal resorption. Importantly, the implant never contacts the shield, leaving a small biological gap that prevents microfractures and allows space for clot stabilization and osseointegration. These refinements make the “Preserving Nature” protocol a safer and more standardized evolution of the socket shield concept, supporting reproducible esthetic and functional results. The minimal palatal remodeling observed (mean 0.18 mm) was clinically negligible. These findings suggest that preserving the palatal root may not be necessary for ridge stability when primary stability is achieved palatally. Standardized adherence to the “Preserving Nature” protocol appears essential for consistent results.

These results support Hürzeler et al.'s [9] original rationale that retaining a buccal root fragment with an intact PDL preserves bundle bone and prevents facial plate collapse. Meta-analyses confirm SST's superiority over conventional immediate implantation, showing reduced marginal bone loss and higher pink-esthetic scores [15, 16, 24]. Within the broader concept of partial extraction therapies (PETs) [22], our buccal-only approach appears to offer predictable three-dimensional stability with minimal morbidity.

The absence of buccal bone loss reinforces the concept that preserving the buccal root fragment and its PDL effectively prevents bundle bone resorption. Limited palatal resorption reflects the lack of preservation on that side, but was minimal and clinically irrelevant.

### 4.1. Clinical Implications

SST should be considered in treatment planning for esthetically demanding anterior sites. The technique preserves hard and soft tissue contours, minimizes surgical invasiveness and morbidity, and may reduce the need for regenerative procedures. It can shorten treatment timelines by eliminating additional augmentation phases.

### 4.2. Future Perspectives

Further research should assess SST under different clinical scenarios (e.g., immediate loading and digital workflows), and compare it with other preservation and augmentation techniques in randomized controlled trials. Although a 3-year follow-up provides meaningful mid-term data, future prospective studies with 5–10 years of follow-up are warranted to confirm the long-term dimensional stability of the alveolar ridge and the biological behavior of the “Preserving Nature” shield over time. Comparative studies of buccal-only versus full-root preservation could clarify any incremental benefits of palatal retention.

### 4.3. Limitations

Several limitations must be acknowledged. The absence of a universally standardized SST protocol may affect reproducibility [25]. Here, strict adherence to the SISS “Preserving Nature” protocol minimized variability. The retrospective design and inclusion criteria—limited to patients with clinically indicated follow-up CBCT—may introduce selection bias and reduce generalizability. The relatively small sample size (*n* = 20) limits the statistical power of this study. However, this reflects the stringent inclusion criteria of a retrospective design based on ethically justified radiographic availability. Despite this limitation, consistent trends across all cases strengthen the reliability of the observed outcomes. CBCT follow-up was constrained by radioprotection norms, preventing volumetric assessments at regular intervals [13]. All cases were treated by a single operator at one center, enhancing consistency but potentially limiting external validity. No comparison group was available for full-root retention, and soft tissue or esthetic outcomes were not assessed.

The inclusion of only patients with a clinically indicated follow-up CBCT, although ethically necessary to minimize radiation exposure, may represent a selection bias and limits the generalizability of the findings

### 4.4. Strengths

Despite these constraints, the study's strengths include a long-term (3-year) follow-up, strict protocol adherence, precise CBCT superimposition with high reproducibility, and homogeneous inclusion of single-rooted teeth in the esthetic zone. The robust statistical approach further supports the validity of the findings. The complete absence of buccal bone loss and minimal palatal remodeling highlight the clinical value of this SST modification in preserving ridge dimensions and reducing the need for additional interventions.

## 5. Conclusions

The present study reinforces the clinical efficacy and reliability of the SST for maintaining alveolar bone volume in the esthetic zone, significantly contributing to the long-term success and patient satisfaction in implant-supported restorations.

The SST described in this study demonstrates significant promise for maintaining alveolar ridge dimensions, particularly in esthetically sensitive regions. Based on these results, the standardized removal of the palatal root and preservation of only the buccal shield, combined with palatal implant anchorage, can be recommended for patients undergoing immediate implant placement who are at risk of substantial alveolar resorption. However, clinicians should adhere strictly to standardized surgical protocols, such as the “Preserving Nature” approach, to optimize outcomes. Future research should focus on long-term prospective randomized controlled trials comparing this approach to other established techniques to further validate its efficacy and establish clearer clinical guidelines.

## Figures and Tables

**Table 1:** Alveolar thickness measurements.

Paz.	Extracted tooth	Alveolar thickness, T0 (mm)	Alveolar thickness, T1 (mm)	Horizontal vestibular bone loss	Horizontal palatal bone loss
1	24	10	8.2	0	1.8
2	45	9.43	9.43	0	0
3	21	9.64	9.64	0	0
4	12	6.45	6.45	0	0
5	23	8.73	7.66	0	1.07
6	21	8.44	8.44	0	0
7	43	8.93	8.93	0	0
8	23	8.86	8.86	0	0
9	11	8.52	8.52	0	0
10	11	7.69	7.69	0	0
11	11	6.54	6.54	0	0
12	14	8.03	8.03	0	0
13	23	11.21	9.93	0	1.28
14	34	7.53	7.53	0	0
15	14	8.80	8.80	0	0
16	25	8.20	8.20	0	0
17	14	8.95	8.95	0	0
18	14	8.35	8.35	0	0
19	46	10.68	10.68	0	0
20	21	9.61	9.61	0	0

**Table 2:** Descriptive statistics.

Parameter	Mean ± SD (mm)	Median (IQR) (mm)	Range (mm)	*p* -Value
Alveolar thickness T0	8.73 ± 1.20	8.77 (8.16–9.47)	6.45–11.21	—
Alveolar thickness T1	8.52 ± 1.07	8.48 (7.94–9.07)	6.45–10.68	—
*Δ* Alveolar thickness (T1 − T0)	−0.21 ± 0.52	0.00 (0.00–0.00)	–1.80–0.00	0.11
Horizontal buccal bone loss	0.00 ± 0.00	0.00 (0.00–0.00)	0.00–0.00	—
Horizontal palatal bone loss	0.18 ± 0.43	0.00 (0.00–0.00)	0.00–1.28	—

**Table 3:** Prevalence of bone loss.

Type of loss	Patients (*n*)	Prevalence (%)
Horizontal buccal	0/20	0
Horizontal palatal	3/20	15

## Data Availability

The data that support the findings of this study are available from the corresponding author upon reasonable request. Due to the retrospective nature of the study and patient confidentiality regulations, the raw CBCT datasets are not publicly available.
